# Reproductive Function in a Population of Young Faroese Men with Elevated Exposure to Polychlorinated Biphenyls (PCBs) and Perfluorinated Alkylate Substances (PFAS)

**DOI:** 10.3390/ijerph15091880

**Published:** 2018-08-30

**Authors:** Maria Skaalum Petersen, Jónrit Halling, Niels Jørgensen, Flemming Nielsen, Philippe Grandjean, Tina Kold Jensen, Pál Weihe

**Affiliations:** 1 Department of Occupational Medicine and Public Health, the Faroes Hospital System, FO-100 Tórshavn, Faroe Islands; jonrith@setur.fo (J.H.); pal@health.fo (P.W.); 2 Center of Health Science, Faculty of Health Sciences, University of the Faroe Islands, FO-100 Tórshavn, Faroe Islands; 3 Faculty of Science and Technology, University of the Faroe Islands, FO-100 Tórshavn, Faroe Islands; 4 Department of Growth and Reproduction, Rigshospitalet, Blegdamsvej 9, 2100 Copenhagen, Denmark; Niels.Joergensen@regionh.dk (N.J.); tkjensen@health.sdu.dk (T.K.J.); 5 Department of Environmental Medicine, University of Southern Denmark, 5000 Odense, Denmark; fnielsen@health.sdu.dk (F.N.); pgrandjean@health.sdu.dk (P.G.); 6 Department of Environmental Health, Harvard T.H. Chan School of Public Health, Boston, MA 02215, USA

**Keywords:** semen quality, reproductive hormones, endocrine disturbing compounds, Faroe Islands

## Abstract

Semen quality may be adversely affected by exposure to environmental chemicals such as polychlorinated biphenyls (PCBs) and perfluorinated alkylate substances (PFASs) that are persistent and may act as endocrine disrupting compounds. The aim of this study was to explore whether PCBs or PFASs exposure were associated with abnormalities in semen quality or reproductive hormones in Faroese men. This population based cross-sectional study includes 263 Faroese men (24–26 years) who delivered a semen sample for assessment of sperm concentration, total sperm count, semen volume, morphology and motility. A blood sample was drawn and analyzed for reproductive hormones, PCBs and PFASs. Exposure to ∑PCBs and perfluorooctane sulfonate (PFOS) was positively associated with sex hormone-binding globulin (SHBG) and luteinizing hormone (LH). In addition, total testosterone (T) was positively associated with ∑PCB. Both PCBs and PFOS appear to lead to increased SHBG, perhaps mediated via the liver. The higher total T associated with PCB may represent a compensatory adaption to elevated SHBG levels to maintain an unchanged free testosterone concentration. The positive association to LH for both PCBs and PFOS may indicate a direct adverse effect on the testosterone producing Leydig cells.

## 1. Introduction

In the Faroe Islands, a North Atlantic fishing community, exposure to persistent organic pollutants (POPs) e.g., polychlorinated biphenyls (PCBs) and perfluorinated alkylate substances (PFASs) are high due to the consumption of the traditional diet that includes whale meat containing PFASs and methylmercury (MeHg) and blubber containing PCBs and other lipophilic substances [1,2,3,4]. The range of exposures is unusually wide, more than 100-fold, with average PCB and MeHg exposures much higher than in any other Western population [5]. While PCB levels among the younger Faroese have decreased by two-thirds in recent years, they are still higher compared with the general US population (e.g., NHANES data) [6]. In contrast, recent data suggest that serum PFASs are similar to those of the U.S. general population [6].

Exposure to PCBs and PFASs is ubiquitous, raising concern about known or potential adverse effects in humans. They are persistent environmental chemicals with long half-lives and endocrine disrupting chemicals (EDCs) that seem to mimic or inhibit the action of endogenous hormones, leading to adverse effects on male reproductive function [7,8].

The effects of PCB exposure on male reproductive function have been investigated in animal and epidemiological studies, though with somewhat conflicting results [8,9,10,11,12,13]. Overall, studies of exposure to PCBs during adulthood indicate some association between PCB and lower sperm motility and, to some extent, decreased sperm DNA chromatin integrity and lower levels of testosterone [8]. 

Few studies have investigated the adverse effects of PFAS exposure on male reproductive function [7,8,14]. Some studies suggest an adverse effect of current and perfluorooctane sulfonate (PFOS) and/or perfluorooctanoic acid (PFOA) exposure on sperm morphology [15,16], motility [16], sperm concentration [17], and reproductive hormone levels [15,18], whereas others report no adverse effects [8,18,19]. 

The aim of the study was therefore to assess associations between current serum concentration of PCBs and PFASs and testicular function among young Faroese men from a population with a wide range of exposure levels. 

## 2. Materials and Methods 

### 2.1. Study Population 

From 2007 to 2009, all young Faroese men (N = 1100) born between January 1981 and December 1984 were invited to participate in this cross-sectional study as previously described [20]. Invitation letters were sent, followed by a phone call to arrange the examination details. A total of 34 men had emigrated, and 43 letters were returned as undeliverable. Thus, 1023 men were invited and of these, 490 could not be reached by phone and they did not respond by mail. Thus, 533 men were reached by phone and 270 declined to participate. Hence, 263 men participated (49% of those reached or 24% of all receiving an invitation letter). Among the participating men, 21 did not provide a semen sample, but they were included in the analysis as they otherwise completed participation in the study. The examinations were carried out during a 24-month period from February 2007. The participating men underwent a physical examination, had a blood sample drawn for reproductive hormone analysis and exposure analysis, delivered a semen sample and answered a questionnaire that included information on previous or current diseases, including any known history of fertility potential and relevant lifestyle factors such as smoking and drinking habits [20].

This study was approved by the Faroese Ethical committee and all participants provided their written informed consent. 

### 2.2. Exposure Assessment

Serum PCB concentrations were determined using solid-phase extraction, and gas chromatographic analysis according to a method previously described [21,22], although the detection of the compounds was performed by a comparable method relying on a TSQ Quantum XLS triple quadrupole mass spectrometer (Thermo Scientific, San José, CA, USA) rather than gas chromatographic system with electron-capture detection. The following PCB congeners were measured: PCB 28, PCB 105, PCB 118, PCB 156, PCB 52, PCB 101, PCB 153, PCB 138 and PCB 180. The results were adjusted for total serum lipid content and reported as μg per gram lipid. The total lipid content was calculated according to Phillips formula [23]. The quality control has been described previously [24]. 

The PCB exposure was estimated as the sum of serum concentrations of the three major PCB congeners 138, 153 and 180 multiplied by 2 [25]. Previous studies in this population have shown that these three PCB congeners represent close to 50% of the total concentration of PCBs [25,26]. The limit of detection was 0.03 µg/L (LOD) for all congeners, which, at a mean lipid concentration of 7.45 g/L, corresponds to 0.004 μg/g lipid. The congeners PCB 118, PCB 138 and PCB 180 were detectable in virtually all samples. 

The PFAS concentrations were measured in all serum samples by online solid-phase extraction followed by high-pressure liquid chromatography with tandem mass spectrometry [3,27]. The analyzes quantified the five major PFASs, i.e., PFOA, PFOS, perfluorohexanesulfonic acid (PFHxS), PFNA (perfluorononanoic acid), and PFDA (perfluorodecanoic acid). Within-batch and between-batch imprecision levels (coefficients of variation) for all five PFASs were <3% and 5–8%, respectively. The quality control of the analysis was confirmed by regular participation in the German-External Quality Assessment Scheme (G-EQUAS) organized by the German Society of Occupational Medicine.

### 2.3. Semen Samples

Semen samples were produced by masturbation in a room close to the semen laboratory. The period of abstinence was recorded. The semen sample was analyzed according to the World Health Organization (WHO) 1999 guidelines [28] modified according to results from a study of inter-observer variation [29]. Semen volume was estimated by weighing the collection tube with the semen sample and subtracting the weight of the empty pre-weighed tube (1 mL semen = 1 g). After liquefaction, the sperm concentration was assessed as previously described [20] using a Bürker–Türk haemocytometer (Paul Marienfeld GmbH & Co. KG, Lauda-Königshofen, Germany). Total sperm count (semen volume x sperm concentration) was also calculated. For sperm motility assessment, 10 μL of well-mixed semen was placed on a clean glass slide kept at 37 °C and covered with a 22 × 22 mm coverslip. The preparation was placed on the heated stage of a microscope at 37 °C and immediately examined at ×400 magnification. The sperm were classified as progressive motile (WHO class AB motility), locally motile (WHO class C motility) or immotile (WHO class D motility). From each semen sample, a smear for morphology evaluation was made, Papanicolaou stained and finally assessed according to ‘strict criteria’ at the Department of Growth and Reproduction at Rigshospitalet (Copenhagen, Denmark) as previously described [20]. 

### 2.4. Reproductive Hormone Analysis

Serum samples were analyzed at University Department of Growth and Reproduction, Rigshospitalet (Copenhagen, Denmark) as previously described [20,24] for serum levels of follicle-stimulating hormone (FSH), luteinizing hormone (LH), sex hormone-binding globulin (SHBG), testosterone (T), estradiol and inhibin B. Free testosterone (FT) was calculated on the basis of the measured serum concentrations of total testosterone and SHBG using the method of Vermeulen et al. assuming a fixed albumin concentration of 0.62 mmol/L [30]. In addition, the ratios inhibin B/FSH, total T/estradiol ratio, total T/LH, and FT/LH were calculated.

### 2.5. Physical Examination 

The study participants underwent a physical examination performed by one of two examiners as previously described [20]. In short, the examination included assessment of body weight and height, the Tanner stage of pubic hair, and any abnormalities of the penis, epididymis or testis including the determination of the location of testis in scrotum. Testicular volumes were determined by palpation using a Prader orchidometer [31].

### 2.6. Statistics 

Outcome variables were semen quality (semen volume, sperm concentration, morphology and motility) and reproductive hormones (FSH, inhibin B, LH, T, FT, estradiol, SHBG, Inhibin B/FSH T/LH, FT/LH, T/estradiol, FT/estradiol) presented as median and interquartile ranges (IQR). The outcome variables were log transformed (all reproductive hormone concentrations and exposure concentrations), cubic root transformed (the sperm concentration, semen volume and total sperm count) or logit transformed (sperm motility) to achieve normal distribution of residuals. Percentages of morphologically normal spermatozoa were close to normally distributed and thus not transformed. The key exposure variables were ∑PCB, PFOA and PFOS; they were log transformed to approach a normal distribution and reduce the relative impact of some very high chemical concentrations. 

First, we evaluated differences in ∑PCB, PFOS and PFOA exposures divided into tertiles (low/median/high) according to characteristics of the men. The crude between-group differences in the characteristics of the men were tested with ANOVA for continuous variables, chi square for categorical variables and t-test when comparing high versus low exposure groups. Correlations between ∑PCB, PFOA and PFAS concentrations were then explored using Spearman correlations. 

Association between semen variables and ∑PCB, PFOA and PFOS levels as continuous variables were tested by linear regression, first crude and then adjusted for confounders; sperm concentration, semen volume and total sperm count were adjusted for period of ejaculation abstinence in hours, the percentages of motile spermatozoa were adjusted for duration from ejaculation to assessment while the reproductive hormones were adjusted for body mass index (BMI) as a continuous variable, smoking (yes/no, including occasional smoking in the yes group), age and hour of day of blood sampling (not included in the analyzes of FSH and LH). 

We examined ∑PCB, the individual PCB congeners and the five measured PFASs as independent exposure indicators, one by one. However, results are presented for ∑PCB which has shown to be representative for the PCB exposure and the two major PFASs, PFOA and PFOS. *p*-values below 5% (two-tailed) were considered statistically significant. Analyzes were performed using SPSS 24.0 (SPSS Inc., Chicago, IL, USA).

## 3. Results

The 263 Faroese men included in this study were between 24 and 26 years old. The median sperm concentration was 37.7 mill/mL and median testosterone level was 19.8 nmol/L. The median ∑PCB was 1.17 µg/g lipid, ranging from 0.1 to 3.4 µg/g lipid. PFOS was by far the most prevalent PFAS followed by PFOA with median PFOA concentration of 2.77 ng/mL, ranging from 0.93 to 20.43 while median PFOS concentration was 19.52 ng/mL, ranging from 4.34 to 72.85. The results of the serum analyzes of the PCB congeners, along with the ∑PCB and related substances and the five PFASs are shown in Supplementary Table S1. All PCB congeners except PCB 28 were highly correlated. Moderate correlations were observed between PFOA and the other PFASs (r from 0.27 up to 0.41) while the correlations between total PFOS and the other PFASs were stronger (r from 0.41 to 0.82). ∑PCB correlated weakly with total PFOS (r = 0.27) and not at all with PFOA (r = −0.04). 

The major descriptive parameters for the study population stratified according to serum levels of exposure are summarized in Table 1. Overall, only few significant differences in main characteristics based on exposure levels were observed. Age was positively associated with PFOA and PFOS levels, BMI was negatively associated with PFOA levels, medication was negatively associated with PFOS levels while SHBG was positively associated both with ∑PCB and PFOA levels. Comparing low versus high exposure, alcohol consumption was negatively associated with PFOS exposure, testis size was positively associated with ∑PCB levels while semen volume was positively associated with PFOA level. 

However, in the regression analysis with exposure as continuous variables and taking confounders into account, the semen variables did not differ according to the ΣPCB, PFAS or PFOA levels as summarized in Table 2. 

There was a significant positive association between the ∑PCB and SHBG, LH, testosterone and the testosterone/estradiol ratio both in the unadjusted and adjusted analyzes (Table 3). Regression coefficients (β) (95% CI) for effect of ∑PCB on SHBG, LH, testosterone and the testosterone/estradiol ratio were 0.05 (0.004–0.09), 0.06 (0.01–0.11), 0.04 (0.004–0.08) and 0.04 (0.01–0.08) respectively, all significant. Adjustment for serum PFOA or PFOS only changed the regression coefficients marginally. Performing the analysis with exposure as groups rather than continuous variables attenuated the associations and only the association between ∑PCB and SHBG and the testosterone/estradiol persisted (data not shown). 

Significant positive associations were observed between PFOS and SHBG and LH, both in the crude and adjusted analyzes. Regression coefficients (β) (95% CI) for effect of PFOS on SHBG and LH were 0.31 (0.02–0.6) and 0.35 (0.02–0.68) respectively. However, after adjustment for ∑PCB, these associations attenuated and became non-significant (β = 0.25, *p* = 0.1 for SHBG and β = 0.26, *p* = 0.1 for LH). Higher PFOA was significantly associated with lower FT/estradiol ratio but not FT or estradiol itself. Adjusting for ∑PCB had very little effect on the associations with PFOA (Table 3).

## 4. Discussion

In this unique population-based study carried out in a population with an unusually wide range of exposure and with average POPs exposures much higher than in any other Western population we found that higher serum PCB and PFOS concentrations were associated with higher levels of SHBG and LH. In addition, higher PCB levels were associated with higher levels of total T and T/E ratio. The altered SHBG levels may be the cause of the higher serum concentration of LH and total T levels, which might reflect a compensatory adaption to the elevated SHBG levels to maintain free testosterone concentrations unchanged. 

We found no association between serum PCB concentration and the semen parameters, which is consistent with previous findings among Faroese fertile men [24] and e.g., Danish men [19]. Also, no association was found between semen variables and any PFAS, in accordance with some other studies [7,32]. Thus, the low semen quality reported among these young men and fertile Faroese men [20] cannot be explained by a direct effect on semen parameters but may be explained by an interference by PCBs and PFAS on the regulation of reproductive hormones [33,34]. A subtle adverse effect on testicular function may well be compensated by altered reproductive hormone stimulation of the testicles, and thus only the latter reveals the toxicity. Another explanation could be that due to the general elevated exposure of the Faroese population to these contaminants, the participating Faroese men were relatively high exposed. Thus, the lack of men with a very low exposure could be an alternative explanation for why we did not see a stronger effect of the current exposure. Also, the men may have been exposed during vulnerable development periods of the testis in utero.

Compared to other studies, the Faroese men had highest PCB153 levels (0.23 ng/g lipid (0.02–6.70 µg/g lipid) followed by Swedish fishermen (0.19 µg/g lipid (0.04–0.15 µg/g lipid) and Greenlandic men (0.17 µg/g lipid (0.05–5.50 µg/g lipid) [35]. The levels were lower among US male partners of infertile couples (0.04 µg/g lipid (0.01–0.36 µg/g lipid) [36]. In contrast, the PFOA and PFOS levels among the Faroese men were comparable or higher than in studies with other populations conducted during the same period [7]. The PFOS levels among Danish young men examined 2008–2009 were lower compared to Faroese levels (PFOS of 7.8 ng/mL vs. 19.15 ng/mL), whereas there was no difference for PFOA levels (3.0 ng/mL vs. 2.77 ng/mL) [19]. However, a US study from 2005–2009 of male partners of couples intending to become pregnant reported median PFOS level of 19.15 ng/mL and PFOA level of 4.6 ng/mL [37], i.e., comparable to the Faroese levels. 

The positive association between PCB and SHBG levels among the Faroese young men is consistent with a previous study of Faroese 14-years old boys where increased SHBG levels were observed at higher PCB exposure levels [38] and, albeit only of borderline significance, in the Faroese fertile men [24]. This association between elevated PCB and SHBG levels has also been observed in other studies [35,39,40,41], although one study did not detect any association [42]. However, none of the studies found concomitants significant positive association with LH and SHBG as this study finds. 

Further, the serum-SHBG concentration was higher among Faroese fertile and young men when compared with Danish men [20,24,43], analyzed in the same laboratory. Also, gonadotropin levels were higher among Faroese men compared with Danes (FSH and LH, *p* < 0.0005) [20] while there was no statistical difference in the levels of inhibin B, T and estradiol (*p* = 0.6). These tendencies indicate deficient testis functions among the Faroese men, which appears to be compensated by the higher levels of gonadotropins. Accordingly, the altered hormone levels could be due to a different endocrine profile among Faroese men as a result of a subtle reduced Leydig cell capacity. 

Due to our study design, we could not document causations. Experimental in vivo studies have, however, shown that elevated exposure to PCBs disrupts and diminishes Leydig cell function in adult rats [44,45,46], and exposing lactating rats to Aroclor 1242, a PCB mixture of varying amounts of mono- through heptachlorinated homologs, caused significant adverse effects on Leydig cell function, reducing the capacity to produce T in response to LH stimulation in adult male offspring [47]. Another study of the toxic effects of Aroclor 1242 on Tm3 Leydig cells suggested that exposure to Aroclor 1242 at high concentrations may result in detrimental effects to Leydig cell homeostasis and impaired steroidogenesis, especially T biosynthesis, by inhibiting two important steroidogenic enzymes [48]. Further, PCBs are known to affect a variety of liver functions [49,50] and PCB-induced hepatic SHBG synthesis could therefore play a possible role contributing to the increased serum-SHBG concentrations observed. This would result in an altered profile of serum concentrations of reproductive hormones compensating for the high SHBG which is evident in this study. 

Our results showed positive significant association between PFOS and SHBG and LH. However, when PCB was included in the model, the association between PFOS and the SHBG and LH attenuated and became only borderline significant. This may indicate that the association is driven by PCB and/or due to reduced statistical power caused by correlation between the exposure variables. The positive association between PFOS and LH has been reported previously in a study of American men from an infertility clinic [18] while other studies pointed in different directions [15,19,51,52]. In accordance with Joensen et al. [15,19], we found a borderline reverse association between free T/LH ratio and PFOS exposure.

One strength of the current study is the analysis of many PCB congeners. Some previous studies relied on PCB153 as the only PCB exposure marker [35,39,40,41]. Although the persistence of PCBs adds validity to our exposure biomarker, mixed PCB exposures may have varied over time, and cross-sectional epidemiological studies are unable to distinguish between actions of the single congeners and other related contaminants and the interrelationships between multiple correlated congeners. This inadequacy often prevents studies from concluding whether one congener is of greater importance than another. Thus, we assume that the sum of major PCB congener concentrations better reflects the overall PCB mixture exposure. However, we did not analyze for co-planar dioxin-like PCBs and thus do not have full information on the dioxin-like activity on the hormones. However, a previous study showed that dioxin exposure in the Faroes is not higher than northern European levels [25]. 

A concern is that our study, as most others, has relied on current serum PFAS and PCB measurements, i.e., not during the most vulnerable developmental stage. Such exposure misclassification will likely result in an underestimation of the true effects of PCB and PFAS exposures [53]. Further, with the cross-sectional nature of the study, reverse causation cannot be excluded. However, the young age of the study participants and the long half-lives of the PCBs and PFASs would argue against reverse causality. A previous study showed that the duration of breastfeeding remained as a significant predictor of serum-PCB concentrations in Faroese adolescents at age 14 years [54]. The data may therefore not only represent the accumulated exposure at the time of clinical examination, but also to some degree past exposures that may reflect vulnerable exposure windows. Further, several comparisons were made without adjustment for multiple testing. However, as most associations tested were based on a priori hypotheses, Bonferroni adjustment would be overly conservative, especially since the statistical tests are not independent [55].

The participation rate was 49%, which may cause a potential selection bias with higher participation of men who had experienced fertility problems. However, these men were relatively young (24–26 years), and the mean age of fathers in a Faroese birth cohort collected in the same time period was 34.8 years [24]. Thus, we would expect that only a minority of men in this age group would be aware of their fertility status, thus limiting the risk of selection bias.

## 5. Conclusions

In this study of young Faroese men, PCBs and PFOS exposure were associated with increased SHBG, perhaps mediated via the liver. The higher total T associated with PCB may represent a compensatory adaption to elevated SHBG levels to maintain an unchanged free testosterone concentration. The positive association to LH for both PCBs and PFOS may indicate a direct adverse effect on the testosterone producing Leydig cells. 

## Figures and Tables

**Table 1:** Characteristics of young Faroese men from the general population born 1981–1984 shown for the entire study population (N = 263) and stratified as high and low exposure according to serum levels below and above median of polychlorinated biphenyls (∑PCBs), perfluorooctanoic acid (PFOA) and perfluorooctane sulfonate (PFOS). Results shown as mean (SD), medians (25–75th percentile) or percentages.

		∑PCB			PFOA			PFOS		
	All Men	Low	Medium	High	Low	Medium	High	Low	Medium	High
	(*n* = 263)	(*n* = 87)	(*n* = 88)	(*n* = 88)	(*n* = 87)	(*n* = 87)	(*n* = 87)	(*n* = 87)	(*n* = 88)	(*n* = 87)
**Physical appearance**										
Age (years)	25.3	25.3	25.4	25.4	***25.1***	***25.3***	***25.6***	***25.1***	***25.4***	***25.6***
	(0.7)	(0.8)	(0.7)	(0.7)	***(0.6)***	***(0.8)***	***(0.7)***	***(0.6)***	***(0.8)***	***(0.7)***
Height (cm)	180	180	179	180	179.0	179.7	179.8	180.2	178.7	179.6
	(6.8)	(6.5)	(7.0)	(7.0)	(6.5)	(7.7)	(6.3)	(6.5)	(7.2)	(6.9)
Weight (kg)	82.6	85.1	80.3	82.3	84.7	81.6	81.4	83.3	82.3	82.2
	(14.0)	(15.4)	(11.6)	(14.5)	(14.8)	(14.3)	(12.7)	(17.1)	(11.5)	(13.2)
BMI (kg/m^2^)	25.5	26.2	25.0	25.4	***26.4***	***25.2***	***25.1***	25.6	25.7	25.4
	(3.7)	(4.2)	(3.2)	(3.5)	***(4.0)***	***(3.6)***	***(3.3)***	(4.4)	(3.3)	(3.2)
Testes size (mL)	21.5	21.3	21.4	21.4	*22.0*	*21.0*	*21*	21.8	21.0	21.1
	(3.5)	(3.5)	(3.8)	(3.2)	*(3.3)*	*(3.6)*	*(3.5)*	(3.7)	(3.2)	(3.5)
**Lifestyle**										
Alcohol (units/week)	4.0	4.0	4.0	5.0	5.0	4.0	5.0	*5.0*	*4.5*	*4.0*
	(1.0–11.0)	(1.0–11.0)	(1.3–14.0)	(1.0–10.0)	(1.0–10.0)	(1.0–11.8)	(1.0–11.0)	*(1.0–15.0)*	*(2.0–10.0)*	*(1.0–9.0)*
Current smokers ^a^, %	51.2	44.2	59.8	49.4	47.1	55.7	50.0	58.1	47.7	47.1
Mother smoked in pregnancy, %	28.8	23.0	21.6	33.8	19.0	32.4	32.9	34.9	27.8	23.2
Taken medication ^b^, %	25.3	27.2	28.9	19.8	25.9	28.2	22.4	**39.2**	**19.3**	**18.3**
**Ever been treated for**										
Cryptorchidism ^c^	3.3	6.3	1.2	2.5	5.0	1.3	3.5	6.5	1.2	2.4
**Ever been diagnosed with**										
Cryptorchidism	9.2	11.5	9.2	6.8	9.3	12.5	5.7	14.0	4.5	9.2
Hypospadias	0.4	0	0	1.2	0	1.1	0	1.1	0	0
Sexual transmitted disease ^d^	9.1	10.3	9.1	8.0	8.0	11.4	8.0	9.2	9.1	9.2
Phimosis	11.1	12.6	9.1	11.6	13.8	11.4	8.2	16.1	10.2	7.1
Varicocele	0.8	1.2	0	1.1	0	1.1	1.2	1.1	0	1.2
**Ever having**										
Caused a pregnancy	25.6	24.1	26.4	26.1	26.4	25.3	25.3	24.4	28.4	24.1
Experienced fertility problems ^e^	4.2	4.7	2.3	5.7	4.6	5.7	2.3	4.6	3.4	4.6
**Semen variables**										
Sperm concentration (mill/mL)	38	36	40	42	39	39	36	37	38	40
	(18–74)	(17–72)	(18–72)	(20–76)	(21–76)	(20–57)	(15–85)	(16–29)	(20–81)	(16–78)
Semen volume (mL)	4.0	3.5	3.9	4.3	3.7	4.0	3.9	*3.6*	*4.0*	*4.2*
	(2.9–5.3)	(2.5–5.4)	(3.1–5.0)	(3.0–5.6)	(2.7–5.5)	(3.0–5.3)	(3.0–5.1)	*(2.5–4.9)*	*(2.8–5.2)*	*(3.0–5.6)*
Total sperm count (mill)	154	118	158	185	149	165	147	133	149	176
	(60–289)	(56–282)	(59–285)	(88–303)	(67–321)	(64–255)	(51–306)	(54–233)	(74–347)	(53–312)
Normal morphology (%)	7.5	6.0	8.5	7.0	8.0	7.5	7.5	7.5	8.0	7.5
	(4.5–10.5)	(4.0–10.0)	(5.5–11.0)	(4.0–10.5)	(5.0–10.5)	(4.0–10.0)	(5.0–11.0)	(4.5–10.5)	(4.0–10.5)	(4.5–10.3)
Motile sperm (%)	74	73	76	74	74	72	77	73	74	77
	(62–84)	(58–85)	(65–84)	(61–81)	(64–83)	(61–83)	(60–86)	(65–81)	(61–83)	(60–86)
Period of abstinence (h)	83	83	76	83	83	84	69	83	73	83
	(60–89)	(60–108)	(59–88)	(60–93)	(64–96)	(60–103)	(47–88)	(61–88)	(59–97)	(59–92)
**Exposure levels**										
∑PCB	0.9	**0.3**	**0.9**	**1.9**	1.0	0.9	0.7	**0.7**	**0.8**	**1.2**
	(0.4–1.6)	**(0.2–0.4)**	**(0.7–1.1)**	**(1.6–2.6)**	(0.4–1.5)	(0.5–1.6)	(0.3–1.6)	**(0.3–1.3)**	**(0.4–1.2)**	**(0.6–2.0)**
PFOA	2.8	2.7	2.8	2.7	**2.0**	**2.8**	**3.8**	**2.4**	**2.7**	**3.3**
	(2.2–3.4)	(2.2–3.5)	(2.2–3.6)	(2.2–3.3)	**(1.7–2.2)**	**(2.6–2.9)**	**(3.4–4.4)**	**(2.0–2.8)**	**(2.2–3.3)**	**(2.6–4.1)**
PFOS	19.5	**18.2**	**19.5**	**21.6**	**17.1**	**18.5**	**23.2**	**13.7**	**19.5**	**28.6**
	(15.4–24.7)	**(14.1–21.7)**	**(14.9–24.3)**	**(16.3–29.7)**	**(13.7–20.7)**	**(14.9–22.7)**	**(19.1–29.2)**	**(12.1–15.4)**	**(18.2–20.9)**	**(24.7–32.3)**
**Reproductive hormones**										
FSH (IU/L)	3.1	3.3	2.7	3.2	2.9	3.3	3.1	2.9	3.0	3.1
	(2.1–4.7)	(2.0–4.9)	(2.1–4.4)	(2.1–5.6)	(1.9–4.2)	(2.3–5.2)	(2.2–4.9)	(2.1–4.9)	(1.9–4.6)	(2.4–4.8)
Inhibin B (pg/mL)	191	194	194	180	184	183	202	194	186	194
	(140–239)	(131–236)	(166–252)	(133–237)	(137–236)	(130–232)	(152–249)	(130–242)	(145–228)	(149–250)
Inhibin B/FSH	57	53	70	56	57	54	60	56	55	57
	(33–112)	(37–109)	(37–114)	(25–113)	(35–119)	(31–99)	(35–111)	(33–127)	(34–112)	(35–109)
LH (IU/L)	4.5	4.4	4.5	4.5	4.6	4.5	4.5	4.2	4.5	4.6
	(3.3–5.5)	(3.1–5.5)	(3.5–5.4)	(3.4–5.9)	(3.5–5.5)	(3.2–5.9)	(3.4–5.4)	(3.2–5.3)	(3.2–5.5)	(3.6–5.7)
T (nmol/L)	19.8	19.0	20.5	19.9	20.4	19.6	19.7	20.1	19.5	20.2
	(16.0–24.3)	(14.0–23.6)	(17.5–24.9)	(16.6–24.4)	(14.9–24.5)	(16.9–25.0)	(16.0–22.6)	(15.6–25.7)	(15.5–22.5)	(16.9–24.0)
FT (pmol/L)	404	408	411	401	402	397	407	390	414	388
	(323–494)	(319–493)	(329–502)	(323–493)	(327–504)	(323–502)	(215–481)	(327–529)	(312–493)	(320–485)
Estradiol (nmol/L)	80	82	78	81	81	78	78	82	76	80
	(66–94)	(67–95)	(62–97)	(66–90)	(67–90	(63–97)	(63–94)	(67–96)	(63–94)	(66–94)
SHBG nmol/L	36	**33**	**37**	**37**	**33**	**37**	**38**	35	34	38
	(27–46)	**(23–47)**	**(29–47)**	**(29–44)**	**(24–42)**	**(30–46)**	**(29–48)**	(27–46)	(25–44)	(30–48)
T/LH	4.7	4.4	4.9	4.5	4.4	4.8	4.5	5.2	4.5	4.2
	(3.4–6.2)	(83.2–6.8)	(3.8–6.0)	(3.4–6.5)	(3.2–5.7)	(3.5–6.3)	(3.6–6.0)	(3.6–7.3)	(3.2–6.1)	(3.4–5.6)
FT/LH	92	89	94	92	90	94	89	*94*	*95*	*86*
	(69–125)	(73–130)	(73–122)	(63–128)	(73–128)	(64–136)	(66–118)	*(77–136)*	*(66–129)*	*(63–118)*
T/estradiol	258	**244**	**265**	**258**	255	259	255	269	246	258
	(210–213)	**(191–309)**	**(226–326)**	**(213–309)**	(217–310)	(208–324)	(204–312)	(205–312)	(204–326)	(219–307)
FT/estradiol	5.2	5.0	5.4	5.1	5.3	5.2	4.9	5.3	5.4	4.9
	(4.2–6.1)	(4.1–5.9)	(4.1–6.2)	(4.3–6.1)	(4.5–6.2)	(4.3–6.2)	(4.0–5.9)	(4.1–6.3)	(4.4–5.9)	(4.2–6.0)

Abbreviation: PCB, polychlorinated biphenyls; ∑PCB = (PCB 138 + PCB 153 + PCB 180) × 2; PFOS = perfluorooctane sulfonic acid; PFOA = perfluorooctanoic acid; FSH, follicle-stimulating hormone; LH, luteinizing hormone; SHBG, sex hormone-binding globulin; T, testosterone; FT, free testosterone. ^a^ Current and occasional smokers; ^b^ Taken any medication 3 months prior to participation in the study; ^c^ Hormonal, surgical or combination; ^d^ Chlamydia, gonorrhoea, warts or herpes; ^e^ Ever had regular intercourse without use of contraception for at least 6 months without partner became pregnant; ANOVA to test for significance between low, medium and high exposure groups (bold text indicates significance); t-test to compare low versus high exposure (italicized text indicates significance); significant at 0.05 level.

**Table 2:** Adjusted regression coefficients (B), 95% confidence intervals (CI) and p values for serum exposure concentrations as predictor for semen quality.

		∑PCB			PFOA			PFOS	
	B	95% CI	*p* ^†^	β	95% CI	*p* ^†^	β	95% CI	*p* ^†^
Sperm concentration (mill/mL) *	0.06	(−0.31–0.44)	0.74	−0.44	(−1.55–0.67)	0.44	0.22	(−0.90–1.34)	0.70
Semen volume (mL) *	0.04	(−0.02–0.10)	0.20	−0.06	(−0.23–0.12)	0.54	0.13	(−0.05–0.31)	0.15
Total sperm count (mill) *	0.29	(−0.32–0.89)	0.35	−0.84	(−2.61–0.94)	0.35	0.82	(−0.98–2.61)	0.37
**Normal morphology (%)**	0.26	(−0.93–1.44)	0.67	−1.49	(−5.06–2.07)	0.41	−0.30	(−3.69–3.09)	0.86
Motile sperm (%) **	0.09	(−0.21–0.39)	0.56	−0.25	(−1.13–0.64)	0.58	0.06	(−0.79–0.91)	0.90

Abbreviation: PCB, polychlorinated biphenyls; ∑PCB = (PCB 138 + PCB 153 + PCB 180) × 2; PFOS = perfluorooctane sulfonic acid; PFOA = perfluorooctanoic acid. The exposure parameters were log transformed while sperm concentration, semen volume and total sperm count were cubic root transformed. * Adjusted for period of abstinence; ** Adjusted for duration from ejaculation to assessment. ^†^ Significant at 0.05 level.

**Table 3:** Regression coefficients (β), 95 CI and p values for serum exposure concentrations as predictor for serum concentrations of reproductive hormones after adjustment for age, body mass index (BMI) groups, current smoking, time of sampling (not for luteinizing hormone (LH) and follicle-stimulating hormone (FSH)).

		∑PCB			PFOA			PFOS	
	β	95 CI	*p* ^†^	β	95 CI	*p* ^†^	β	95 CI	*p* ^†^
**FSH (IU/L)**	0.01	(−0.06–0.09)	0.77	0.32	(−0.19–0.83)	0.22	0.23	(−0.25–0.72)	0.34
**Inhibin B (pg/mL)**	0.05	(−0.04–0.14)	0.27	0.07	(−0.56–0.69)	0.83	0.09	(−0.51–0.69)	0.77
**Inhibin B/FSH**	0.04	(−0.11–0.19)	0.61	−0.09	(−0.54–0.36)	0.69	−0.08	(−0.51–0.35)	0.72
**LH (IU/L)**	**0.06**	**(0.01–0.11)**	**0.02**	−0.11	(−0.46–0.24)	0.54	**0.35**	**(0.02–0.68)**	**0.04**
**T (nmol/L)**	**0.04**	**(0.004–0.08)**	**0.03**	−0.11	(−0.39–0.16)	0.42	0.11	(−0.16–0.38)	0.41
**FT (pmol/L)**	0.02	(−0.02–0.07)	0.24	−0.28	(−0.56–0.002)	0.05	−0.03	(−0.30–0.25)	0.85
**Estradiol (nmol/L)**	0.00	(−0.04–0.03)	0.94	−0.01	(−0.25–0.23)	0.93	0.07	(−0.15–0.30)	0.52
**SHBG nmol/L**	**0.05**	**(0.004–0.09)**	**0.03**	0.23	(−0.08–0.53)	0.14	**0.31**	**(0.02–0.60)**	**0.04**
**T/LH**	−0.02	(−0.08–0.04)	0.55	0.00	(−0.19–0.19)	1.00	−0.02	(−0.30–0.06)	0.21
**FT/LH**	−0.04	(−0.10–0.03)	0.23	−0.07	(−0.26–0.12)	0.46	−0.18	(−0.36–0.01)	0.06
**T/estradiol**	**0.05**	**(0.01–0.08)**	**0.01**	−0.05	(−0.15–0.06)	0.41	0.02	(−0.09–0.12)	0.78
**FT/estradiol**	0.03	(−0.01–0.06)	0.12	**−0.12**	**(−0.21–0.02)**	**0.02**	−0.05	(−0.14–0.62)	0.35

Abbreviation: PCB, polychlorinated biphenyls; ∑PCB = (PCB 138 + PCB 153 + PCB 180) × 2; FSH, follicle-stimulating hormone; LH, luteinizing hormone; SHBG, sex hormone-binding globulin; T, testosterone; FT, free testosterone. All parameters are log transformed. ^†^ Significant at 0.05 level (bold text indicates significance).

## Supplementary Materials

The following are available online at http://www.mdpi.com/1660-4601/15/9/1880/s1, Table S1: Supplementary Table 1. Serum concentrations (µg/g lipid) of polychlorinated biphenyls (PCBs), persistent organic pollutants (POPs) and perfluorinated alkylate substances (PFAS). Results shown as medians and range.

## References

[1] Heilmann C., Budtz-Jorgensen E., Nielsen F., Heinzow B., Weihe P., Grandjean P. (2010). Serum concentrations of antibodies against vaccine toxoids in children exposed perinatally to immunotoxicants. Environ. Health Perspect..

[2] Heilmann C., Grandjean P., Weihe P., Nielsen F., Budtz-Jorgensen E. (2006). Reduced antibody responses to vaccinations in children exposed to polychlorinated biphenyls. PLoS Med..

[3] Grandjean P., Andersen E.W., Budtz-Jorgensen E., Nielsen F., Molbak K., Weihe P., Heilmann C. (2012). Serum vaccine antibody concentrations in children exposed to perfluorinated compounds. JAMA.

[4] Weihe P., Joensen H.D. (2012). Dietary recommendations regarding pilot whale meat and blubber in the Faroe Islands. Int. J. Circumpolar Health.

[5] Longnecker M.P., Wolff M.S., Gladen B.C., Brock J.W., Grandjean P., Jacobson J.L., Korrick S.A., Rogan W.J., Weisglas-Kuperus N., Hertz-Picciotto I. (2003). Comparison of polychlorinated biphenyl levels across studies of human neurodevelopment. Environ. Health Perspect..

[6] (2015). Fourth National Report on Human Exposure to Environmental Chemicals.

[7] Bach C.C., Vested A., Jorgensen K.T., Bonde J.P., Henriksen T.B., Toft G. (2016). Perfluoroalkyl and polyfluoroalkyl substances and measures of human fertility: A systematic review. Crit. Rev. Toxicol..

[8] Vested A., Giwercman A., Bonde J.P., Toft G. (2014). Persistent organic pollutants and male reproductive health. Asian J. Androl..

[9] Meeker J.D., Hauser R. (2010). Exposure to polychlorinated biphenyls (PCBs) and male reproduction. Syst. Biol. Reprod. Med..

[10] Toft G., Hagmar L., Giwercman A., Bonde J.P. (2004). Epidemiological evidence on reproductive effects of persistent organochlorines in humans. Reprod. Toxicol..

[11] Carpenter D.O. (2006). Polychlorinated biphenyls (PCBs): Routes of exposure and effects on human health. Rev. Environ. Health.

[12] (2000). ATSDR. http://www.atsdr.cdc.gov/toxprofiles/tp.asp?id=142&tid=26.

[13] Cook M.B., Trabert B., McGlynn K.A. (2011). Organochlorine compounds and testicular dysgenesis syndrome: Human data. Int. J. Androl..

[14] Foresta C., Tescari S., di Nisio A. (2018). Impact of perfluorochemicals on human health and reproduction: A male’s perspective. J. Endocrinol. Investig..

[15] Joensen U.N., Bossi R., Leffers H., Jensen A.A., Skakkebaek N.E., Jorgensen N. (2009). Do perfluoroalkyl compounds impair human semen quality?. Environ. Health Perspect..

[16] Toft G., Jonsson B.A., Lindh C.H., Giwercman A., Spano M., Heederik D., Lenters V., Vermeulen R., Rylander L., Pedersen H.S. (2012). Exposure to perfluorinated compounds and human semen quality in Arctic and European populations. Hum. Reprod..

[17] Vested A., Ramlau-Hansen C.H., Olsen S.F., Bonde J.P., Stovring H., Kristensen S.L., Halldorsson T.I., Rantakokko P., Kiviranta H., Ernst E.H. (2014). In utero exposure to persistent organochlorine pollutants and reproductive health in the human male. Reproduction.

[18] Raymer J.H., Michael L.C., Studabaker W.B., Olsen G.W., Sloan C.S., Wilcosky T., Walmer D.K. (2012). Concentrations of perfluorooctane sulfonate (PFOS) and perfluorooctanoate (PFOA) and their associations with human semen quality measurements. Reprod. Toxicol..

[19] Joensen U.N., Veyrand B., Antignac J.P., Jensen M.B., Petersen J.H., Marchand P., Skakkebæk N.E., Andersson A.M., Le Bizec B., Jørgensen N. (2013). PFOS (perfluorooctanesulfonate) in serum is negatively associated with testosterone levels, but not with semen quality, in healthy men. Hum. Reprod..

[20] Halling J., Petersen M.S., Jorgensen N., Jensen T.K., Grandjean P., Weihe P. (2013). Semen quality and reproductive hormones in Faroese men: A cross-sectional population-based study of 481 men. BMJ Open.

[21] Petersen M.S., Halling J., Damkier P., Nielsen F., Grandjean P., Weihe P., Brøsen K. (2006). Caffeine N_3_-demethylation (CYP_1_A_2_) in a population with an increased exposure to polychlorinated biphenyls. Eur. J. Clin. Pharmacol..

[22] Grandjean P., Weihe P., Nielsen F., Heinzow B., Debes F., Budtz-Jorgensen E. (2012). Neurobehavioral deficits at age 7 years associated with prenatal exposure to toxicants from maternal seafood diet. Neurotoxicol. Teratol..

[23] Phillips D.L., Pirkle J.L., Burse V.W., Bernert J.T., Henderson L.O., Needham L.L. (1989). Chlorinated hydrocarbon levels in human serum: Effects of fasting and feeding. Arch. Environ. Contam. Toxicol..

[24] Petersen M.S., Halling J., Weihe P., Jensen T.K., Grandjean P., Nielsen F., Jørgensen N. (2015). Spermatogenic capacity in fertile men with elevated exposure to polychlorinated biphenyls. Environ. Res..

[25] Grandjean P., Weihe P., Needham L.L., Burse V.W., Patterson D.G., Sampson E.J., Jorgensen P.J., Vahter M. (1995). Relation of a seafood diet to mercury, selenium, arsenic, and polychlorinated biphenyl and other organochlorine concentrations in human milk. Environ. Res..

[26] Steuerwald U., Weihe P., Jorgensen P.J., Bjerve K., Brock J., Heinzow B., Budtz-Jørgensen E., Grandjean P. (2000). Maternal seafood diet, methylmercury exposure, and neonatal neurologic function. J. Pediatr..

[27] Haug L.S., Thomsen C., Becher G. (2009). A sensitive method for determination of a broad range of perfluorinated compounds in serum suitable for large-scale human biomonitoring. J. Chromatogr. A.

[28] World Health Organization (1999). Laboratory Maual for Examination of Human Semen and Semen-Cervicl Mucus Interaction.

[29] Jorgensen N., Auger J., Giwercman A., Irvine D.S., Jensen T.K., Jouannet P., Keiding N., Bon C.L., MacDonald E., Pekuri A.M. (1997). Semen analysis performed by different laboratory teams: An intervariation study. Int. J. Androl..

[30] Vermeulen A., Verdonck L., Kaufman J.M. (1999). A critical evaluation of simple methods for the estimation of free testosterone in serum. J. Clin. Endocrinol. Metab..

[31] Lenz S., Giwercman A., Elsborg A., Cohr K.H., Jelnes J.E., Carlsen E., Skakkebœk N.E. (1993). Ultrasonic testicular texture and size in 444 men from the general population: Correlation to semen quality. Eur. Urol..

[32] Vested A., Ramlau-Hansen C.H., Olsen S.F., Bonde J.P., Kristensen S.L., Halldorsson T.I., Becher G., Haug L.S., Ernst E.H., Toft G. (2013). Associations of in utero exposure to perfluorinated alkyl acids with human semen quality and reproductive hormones in adult men. Environ. Health Perspect..

[33] Bonefeld-Jorgensen E.C., Andersen H.R., Rasmussen T.H., Vinggaard A.M. (2001). Effect of highly bioaccumulated polychlorinated biphenyl congeners on estrogen and androgen receptor activity. Toxicology.

[34] Kjeldsen L.S., Bonefeld-Jorgensen E.C. (2013). Perfluorinated compounds affect the function of sex hormone receptors. Environ. Sci. Pollut. Res. Int..

[35] Giwercman A.H., Rignell-Hydbom A., Toft G., Rylander L., Hagmar L., Lindh C., Pedersen H.S., Ludwicki J.K., Lesovoy V., Shvets M. (2006). Reproductive hormone levels in men exposed to persistent organohalogen pollutants: A study of inuit and three European cohorts. Environ. Health Perspect..

[36] Hauser R., Chen Z., Pothier L., Ryan L., Altshul L. (2003). The relationship between human semen parameters and environmental exposure to polychlorinated biphenyls and p,p’-DDE. Environ. Health Perspect..

[37] Louis G.M., Chen Z., Schisterman E.F., Kim S., Sweeney A.M., Sundaram R., Lynch C.D., Gore-Langton R.E., Barr D.B. (2015). Perfluorochemicals and human semen quality: The LIFE study. Environ. Health Perspect..

[38] Grandjean P., Gronlund C., Kjaer I.M., Jensen T.K., Sorensen N., Andersson A.M., Juul A., Skakkebæk N.E., Budtz-Jørgensen E., Weihe P. (2012). Reproductive hormone profile and pubertal development in 14-year-old boys prenatally exposed to polychlorinated biphenyls. Reprod. Toxicol..

[39] Haugen T.B., Tefre T., Malm G., Jonsson B.A., Rylander L., Hagmar L., Bjørsvik C., Henrichsen T., Sæther T., Figenschau Y. (2011). Differences in serum levels of CB-153 and p,p’-DDE, and reproductive parameters between men living south and north in Norway. Reprod. Toxicol..

[40] Bonde J.P., Toft G., Rylander L., Rignell-Hydbom A., Giwercman A., Spano M., Manicardi G.C., Bizzaro D., Ludwicki J.K., Zvyezday V. (2008). Fertility and markers of male reproductive function in Inuit and European populations spanning large contrasts in blood levels of persistent organochlorines. Environ. Health Perspect..

[41] Richthoff J., Rylander L., Jonsson B.A., Akesson H., Hagmar L., Nilsson-Ehle P., Stridsberg M., Giwercman A. (2003). Serum levels of 2,2’,4,4’,5,5’-hexachlorobiphenyl (CB-153) in relation to markers of reproductive function in young males from the general Swedish population. Environ. Health Perspect..

[42] Hagmar L., Bjork J., Sjodin A., Bergman A., Erfurth E.M. (2001). Plasma levels of persistent organohalogens and hormone levels in adult male humans. Arch. Environ. Health.

[43] Jorgensen N., Joensen U.N., Jensen T.K., Jensen M.B., Almstrup K., Olesen I.A., Juul A., Andersson A.M., Carlsen E., Petersen J.H. (2012). Human semen quality in the new millennium: A prospective cross-sectional population-based study of 4867 men. BMJ Open.

[44] Murugesan P., Balaganesh M., Balasubramanian K., Arunakaran J. (2007). Effects of polychlorinated biphenyl (Aroclor 1254) on steroidogenesis and antioxidant system in cultured adult rat Leydig cells. J. Endocrinol..

[45] Kovacevic R., Vojinovic-Miloradov M., Teodorovic I., Andric S. (1995). Effect of PCBs on androgen production by suspension of adult rat Leydig cells in vitro. J. Steroid Biochem. Mol. Biol..

[46] Murugesan P., Kanagaraj P., Yuvaraj S., Balasubramanian K., Aruldhas M.M., Arunakaran J. (2005). The inhibitory effects of polychlorinated biphenyl Aroclor 1254 on Leydig cell LH receptors, steroidogenic enzymes and antioxidant enzymes in adult rats. Reprod. Toxicol..

[47] Kim I.S., Ariyaratne H.B., Mendis-Handagama S.M.C. (2001). Effects of continuous and intermittent exposure of lactating mothers to aroclor 1242 on testicular steroidogenic function in the adult male offspring. Tissue Cell.

[48] Aydin Y., Erkan M. (2017). The toxic effects of polychlorinated biphenyl (Aroclor 1242) on Tm3 Leydig cells. Toxicol. Ind. Health.

[49] European Food Safety Authority (EFSA) (2005). Opinion of the scientific panel on contaminants in the food chain on a request from the Commission related to the presence of non-dioxin-like polychlorinated biphenyls (PCB) in feed and food. EFSA J..

[50] Kumar J., Lind L., Salihovic S., van Bavel B., Ingelsson E., Lind P.M. (2014). Persistent organic pollutants and liver dysfunction biomarkers in a population-based human sample of men and women. Environ. Res..

[51] Olsen G.W., Gilliland F.D., Burlew M.M., Burris J.M., Mandel J.S., Mandel J.H. (1998). An epidemiologic investigation of reproductive hormones in men with occupational exposure to perfluorooctanoic acid. J. Occup. Environ. Med..

[52] Den Hond E., Tournaye H., de Sutter P., Ombelet W., Baeyens W., Covaci A., Cox B., Nawrot T.S., Van Larebeke N., D’Hooghe T. (2015). Human exposure to endocrine disrupting chemicals and fertility: A case-control study in male subfertility patients. Environ. Int..

[53] Grandjean P., Budtz-Jorgensen E. (2010). An ignored risk factor in toxicology: The total imprecision of exposure assessment. Pure Appl. Chem..

[54] Barr D.B., Weihe P., Davis M.D., Needham L.L., Grandjean P. (2006). Serum polychlorinated biphenyl and organochlorine insecticide concentrations in a Faroese birth cohort. Chemosphere.

[55] Rothman K.J. (2014). Six persistent research misconceptions. J. Gen. Intern. Med..

