# Supplementary material for: Reproductive Function in a Population of Young Faroese Men with Elevated Exposure to Polychlorinated Biphenyls (PCBs) and Perfluorinated Alkylate Substances (PFAS)
Source: Int J Environ Res Public Health. 2018 Aug 30;15(9):1880. doi: 10.3390/ijerph15091880 (PMC6165232; doi:10.3390/ijerph15091880)
Supplement: Supplementary file 1 [file ijerph-15-01880-s001.pdf]

Supplementary table 1. Serum concentrations (µg/g lipid) of polychlorinated biphenyls (PCBs), persistent organic pollutants (POPs) and perfluorinated alkylate substances (PFAS). Results shown as medians and range.

	**All men (=263)**		
**Exposure**	% >LOD	Median	Range
**PCB28**	92	0.01	0.002-0.02
**PCB52**	66	0.007	0.001-0.01
**PCB101**	54	0.007	0.0007-0.02
**PCB118**	91	0.04	0.002-0.15
**PCB153**	100	0.230	0.02-0.67
**PCB105**	77	0.009	0.001-0.02
**PCB156**	75	0.008	0.001-0.02
**PCB180**	99	0.16	0.009-0.50
**PCB138**	99	0.19	0.01-0.53
**∑PCB**	100	1.17	0.1-3.4
**PFOA**	100	2.77	0.93-20.43
**PFHxS**	100	0.81	0.18-2.46
**PFNA**	100	1.49	0.61-18.10
**PFDA**	100	0.52	0.16-4.59
**brPFOS**	100	7.36	0.86-27.73
**nPFOS**	100	12.16	3.48-45.12
**PFOS**	100	19.52	4.34-72.85

Abbreviation: LOD= limit of detection; CI= confidence interval; ΣPCB=(PCB 138 + PCB 153 + PCB 180)*2; PCB= polychlorinated biphenyls; HCB=hexachlorobenzen; betaHCH=beta hexachlorocyclohexan; pp-DDE=p,p'-Dichlorodiphenyldichloroethylene; PFOS=perfluorooctane sulfonic acid;PFOA=perfluorooctanoic acid; PFHxS=perfluorohexanesulfonic acid, PFNA=perfluorononanoic acid and PFDA =perfluorodecanoic acid).
